# A descriptive analysis of non-Cochrane child-relevant systematic reviews published in 2014

**DOI:** 10.1186/s12874-018-0562-2

**Published:** 2018-10-01

**Authors:** Michelle Gates, Sarah A Elliott, Cydney Johnson, Denise Thomson, Katrina Williams, Ricardo M Fernandes, Lisa Hartling

**Affiliations:** 1 Alberta Research Centre for Health Evidence, Department of Pediatrics, University of Alberta, Edmonton, AB Canada; 2 Cochrane Child Health, Department of Pediatrics, University of Alberta, Edmonton, AB Canada; 3 School of Pediatrics, Royal Children’s Hospital, Melbourne, VIC Australia; 4 Department of Pediatrics, Santa Maria Hospital, Lisbon Academic Center, Lisbon, Portugal; 5 Clinical Pharmacology and Therapeutics, Instituto de Medicina Molecular, Universidade de Lisboa, Lisbon, Portugal; 6 School of Pediatrics, Royal Children’s Hospital, 50 Flemington Road, Parkville, VIC 20152 Australia; 7 Department of Pediatrics, Hospital Santa Maria (CHLN), Lisbon Academic Center, Lisbon, Portugal

**Keywords:** Systematic review, Pediatrics, Child, Methods

## Abstract

**Background:**

Consumers, clinicians, policymakers and researchers require high quality evidence to guide decision-making in child health. Though Cochrane systematic reviews (SRs) are a well-established source of evidence, little is known about the characteristics of non-Cochrane child-relevant SRs. To complement published descriptions of Cochrane SRs, we aimed to characterize the epidemiologic, methodological, and reporting qualities of non-Cochrane child-relevant SRs published in 2014.

**Methods:**

English-language child-relevant SRs of quantitative primary research published outside the Cochrane Library in 2014 were eligible for this descriptive analysis. A research librarian searched MEDLINE, CINAHL, Web of Science, and PubMed in August 2015. A single reviewer screened articles for inclusion; a second verified the excluded studies. Reviewers extracted: general characteristics of the review; included study characteristics; methodological approaches. We performed univariate analyses and presented the findings narratively.

**Results:**

We identified 1598 child-relevant SRs containing a median (IQR) 19 (11, 33) studies. These originated primarily from high-income countries (*n* = 1247, 78.0%) and spanned 47 of the 53 Cochrane Review Groups. Most synthesized therapeutic (*n* = 753, 47.1%) or epidemiologic (*n* = 701, 43.8%) evidence. Though 39.3% (*n* = 628) of SRs included evidence related to children only, few were published in pediatric-specific journals (*n* = 283, 17.7%). Reporting quality seemed poor based on the items we assessed; few reviews mentioned an a-priori protocol (*n* = 246, 15.4%) or registration (*n* = 111, 6.9%), and only 23.4% (*n* = 374) specified a primary outcome. Many SRs relied solely on evidence from non-RCTs (*n* = 796, 49.8%). Less than two-thirds (*n* = 953, 59.6%) appraised the quality of included studies and assessments of the certainty of the body of evidence were rare (*n* = 102, 6.4%).

**Conclusions:**

Child-relevant Cochrane SRs are a known source of high quality evidence in pediatrics. There exists, however, an abundance of evidence from non-Cochrane SRs that may be complementary. Our findings show that high-quality non-Cochrane SRs may not be practical nor easy for knowledge users to find. Improvements are needed to ensure that evidence syntheses published outside of the Cochrane Library adhere to the high standard of conduct and reporting characteristic of Cochrane SRs.

**Electronic supplementary material:**

The online version of this article (10.1186/s12874-018-0562-2) contains supplementary material, which is available to authorized users.

## Background

Consumers, clinicians, policymakers and researchers require up-to-date evidence to guide decision-making, but keeping up with the rapid accumulation of primary research is a challenge. [1] Systematic reviews (SRs) provide a transparent synthesis of all available evidence on a given research question. [2] They are used to inform healthcare decisions, [3] evidence-based practice guidelines, [4] and can facilitate the identification of knowledge gaps, thereby reducing research waste by guiding where new research is most needed. [5, 6] However, findings of SRs that are outdated, methodologically flawed, or incompletely reported are of limited use. [7]

Although clear standards for conduct and reporting are publicly available to authors and widely endorsed, [8] many reviews fall short [9] of best-practice standards. [10, 11] In a comparison of biomedical SRs published in 2004 [12] and 2014, [9] Page et al. (2016) warned that the rise in popularity of evidence syntheses has been accompanied by a proliferation of substandard SRs. [9] Performance bonuses for publication [13] and the rise of predatory publishers [14, 15] are two suggested contributors. These poorly-conducted reviews run the risk of propagating biased, misleading, or harmful conclusions. [16]

Periodically characterizing all available SRs can increase awareness of the areas in need of improvement. Given the shortage of child-specific evidence for healthcare decision making, [17–19] the need for high quality evidence syntheses in pediatrics is clear. In 2009 [20] and 2013, [21] authors from our research group undertook descriptive analyses of all child-relevant SRs in the Cochrane Library. Reporting quality of the SRs was highly variable and many were out-of-date. [21] Few focused solely on children or conducted child-specific subgroup analyses. [21]

No similar efforts have been made to characterize child-relevant non-Cochrane SRs. Cochrane reviews represent a small minority [9] of the mounting number of available reviews, [16] and are known to differ from non-Cochrane reviews in terms of scope and reporting quality. [9, 12] Thus, the knowledge gap in terms of the quality and quantity of non-Cochrane evidence available in the field of pediatrics is substantial. We aimed to identify all non-Cochrane child-relevant SRs published over 1 year (2014) and describe the characteristics of these SRs, their included studies, and methodological approaches.

## Methods

### Design

We conducted a cross-sectional descriptive study of all SRs on any child-relevant topic published outside of the Cochrane Library over 1 year. Ethics approval was not required because there were no human participants.

### Eligibility criteria

English language child-relevant SRs of quantitative primary research published outside of the Cochrane Library in 2014 were eligible. We used Cochrane [10] standards to define SRs for inclusion, with some flexibility (e.g., we did not require a quality appraisal) to account for the fact that many SRs published outside of the Cochrane library might not meet their stringent criteria nor adhere to published reporting standards such as the PRISMA statement (Preferred Reporting Items for Systematic reviews and Meta-Analyses). [11, 22] Thus, we considered SRs to be those publications that reported an explicitly defined methodology, including: [10, 11, 22] (a) a comprehensive search (i.e., at least two databases or one database and a supplementary method such as hand-searching); (b) a description of study selection, including the number of records identified via the search and primary studies retained for the review; (c) predefined eligibility criteria; (d) a description of the data extraction methodology and/or items extracted; and (e) a summary of findings. We excluded Cochrane SRs (including duplicate or summary publications published outside the Cochrane Library), theses and dissertations, and all other types of reviews (i.e., rapid reviews, scoping reviews, iterative reviews, reviews of qualitative studies, mixed methods reviews, overviews of reviews).

We defined ‘child-relevant’ SRs as those that intended to include children (0 to 18 years) or presented at least one outcome pertaining to children. [20, 21] This included interventions on adults that were intended to improve the health of children (e.g., smoking cessation programs for post-partum mothers [23]), and reviews including both children and adults, even when child-specific subgroup analyses were not conducted. We included SRs on neonates, those that reported on outcomes measured in adults as a result of exposures occurring in childhood (e.g., post-traumatic stress resulting from child abuse [24]) and those relating to pregnancy and breastfeeding with child-relevant outcomes (e.g., birth weight, [25] congenital malformations, [26] outcomes in the child resulting from breastfeeding [27]). We excluded SRs reporting only fetal outcomes, preterm delivery or stillbirth.

### Literature search

A research librarian developed a search strategy to locate child-relevant SRs that included subject headings related to the pediatric population, and SRs (Additional file 1). Searches were undertaken on August 17, 2015 in Ovid MEDLINE(R) In-Process & Other Non-Indexed Citations and Ovid Medline(R) 1946 to present, CINAHL via EBSCOhost, the Web of Science Core Collection, and PubMed. We applied a methodological search filter to restrict the search results to SRs. [28] The searches were also restricted to English language SRs in humans published from January 1 to December 31, 2014.

### Selection

We employed a two-stage selection process. First, one reviewer screened the titles and abstracts of all records and classified them as ‘include’, ‘exclude’ or ‘unsure’. A second reviewer screened all ‘excludes’. We then retrieved the full text of any record categorized as ‘include’ or ‘unsure’ by either reviewer. We followed the same process for full text screening. Disagreements were resolved by discussion or the involvement of a third reviewer.

### Data extraction

One reviewer developed a data extraction form in Microsoft Office Excel (v. 2016, Microsoft Corporation, Redmond, WA). Three reviewers independently pilot tested the form on a sample of 5 to 10 SRs. Then, a single reviewer independently extracted data from the SRs. The three reviewers met regularly to resolve uncertainties.

We extracted three categories of data, similar to previous descriptive analyses of child-relevant Cochrane SRs, [20, 21] to optimize comparability: 1) general SR characteristics i.e., country of the corresponding author and income classification (World Bank [29]), type of journal, type of review question (i.e., therapeutic [treatment and prevention], epidemiological [prevalence, association between exposure and outcome, genetic association reviews], diagnostic/prognostic [accuracy of diagnostic tests, prognostic factors, clinical prediction rules], or other [cost-effectiveness, psychometric properties of measurement tools, others that do not fit any group]), [9] topic of the review based on existing Cochrane Review Groups (CRGs, to allow comparison in the quantity of evidence by topic area), [30] whether the SR was an update, sources of funding, mention of published or registered review protocol and/or registration within a SR registry; 2) characteristics of the included primary studies i.e., primary study designs sought and included, the number of reports of studies and number of human participants included, and the type of participants included; and 3) methodological approaches i.e., statement of an objective and primary outcome, quality appraisal of the included studies, assessment of certainty of body of evidence (defined as “extent of our confidence that the estimates of effect are correct” [31]), and synthesis method. Additional file 2 shows detailed information about the data extraction items. We extracted the data as reported in the SRs, and did not consult authors, search for published protocols or registration, or use other methods to acquire additional information.

We examined the agreement between reviewers via duplicate extraction of a random sample of 10% (*n* = 160/1598) of the SRs. One of the reviewers involved in data extraction verified all data contained in any data item where agreement was < 85% in the random sample. Any other noted discrepancies were also corrected.

### Data analysis

We performed univariate analyses in Excel to describe the general characteristics of the SRs, their included studies and methodological approaches. We calculated counts and frequencies for categorical data, and medians and interquartile ranges for continuous data. We presented the findings narratively.

## Results

### Selected sample of systematic reviews

**Fig. 1:**
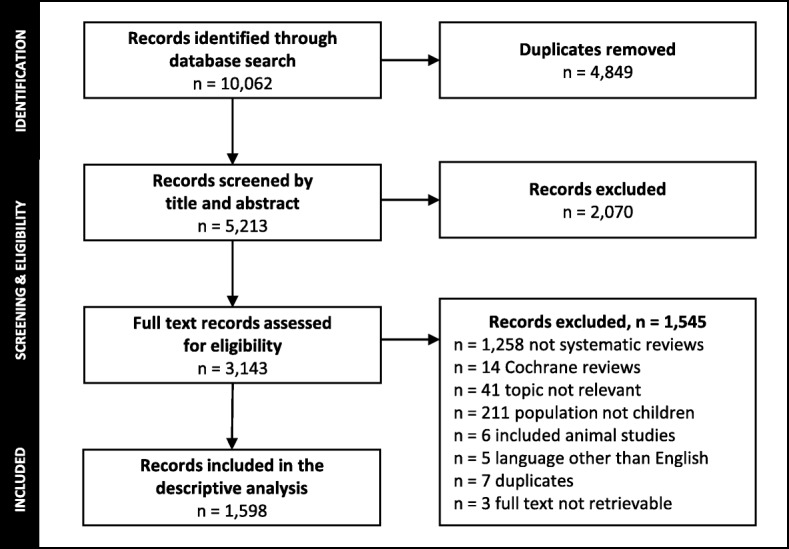
Flow of records through the selection process

We retrieved 5213 unique records via the search and excluded 2070 during title and abstract screening. We inspected the full text of 3143 records and excluded 1545 (Fig. 1). Most full text exclusions were related to publications not meeting our definition of a SR (*n* = 1258/1545, 81.4%). The final sample included 1598 child-relevant SRs containing a median (IQR) 19 (11 to 33) reports of studies and 2134 (648 to 13,079) participants.

### Characteristics of the overall sample

Tables 1, 2 and 3 show the SR, included primary studies, and methodological characteristics of the sample, overall and by type of review question. The SRs originated almost exclusively from high (*n* = 1247, 78.0%) and upper-middle (*n* = 317, 19.8%) income countries. The USA (*n* = 384, 24.0%), China (*n* = 201, 12.6%), the UK (n = 201, 12.6%), Canada (*n* = 150, 9.4%), and Australia (*n* = 136, 8.5%) were the top child-relevant SR-producing countries.

**Table 1:** Characteristics of non-Cochrane child-relevant systematic reviews published in 2014, overall and by type of review question

Review characteristics	Overall	Type of review question^a^			
		Therapeutic	Epidemiology	Diagnostic/Prognostic	Other
Total number of systematic reviews (%)	1598 (100.0)	753 (47.1)	701 (43.8)	91 (5.7)	53 (3.3)
Corresponding author: continent, n (%)					
Asia	299 (18.7)	123 (16.3)	148 (21.1)	25 (27.5)	3 (5.7)
Africa	24 (1.5)	11 (1.5)	11 (1.6)	1 (1.1)	1 (1.9)
North America	534 (33.4)	287 (38.1)	199 (28.4)	30 (33.0)	18 (34.0)
South America	65 (4.1)	25 (3.3)	37 (5.3)	2 (2.2)	1 (1.9)
Europe	535 (33.5)	255 (33.9)	232 (33.1)	26 (28.6)	22 (41.5)
Oceania	141 (8.8)	52 (6.9)	74 (10.6)	7 (7.7)	8 (15.1)
Corresponding author: country income level (World Bank), n (%)					
High	1247 (78.0)	610 (81.0)	524 (74.8)	64 (70.3)	49 (92.5)
Upper middle	317 (19.8)	126 (16.7)	163 (23.3)	25 (27.5)	3 (5.7)
Lower middle	27 (1.7)	15 (2.0)	9 (1.3)	2 (2.2)	1 (1.9)
Low	7 (0.4)	2 (0.3)	5 (0.7)	0 (0.0)	0 (0.0)
Journal type, n (%)					
General medical	73 (4.6)	28 (3.7)	43 (6.1)	1 (1.1)	1 (1.9)
Specialty medical	1137 (71.2)	535 (71.0)	503 (71.8)	67 (73.6)	32 (60.4)
General pediatric	110 (6.9)	63 (8.4)	35 (5.0)	8 (8.8)	4 (7.5)
Specialty pediatric	173 (10.8)	87 (11.6)	68 (9.7)	6 (6.6)	12 (22.6)
Other^b^	105 (6.6)	40 (5.3)	52 (7.4)	9 (9.9)	4 (7.5)
Sources of funding^c^, n (%)					
Academic	228 (14.3)	96 (12.7)	118 (16.8)	9 (9.9)	5 (9.4)
Government	557 (34.9)	239 (31.7)	265 (37.7)	37 (40.7)	16 (30.2)
Industry	53 (3.3)	23 (3.1)	22 (3.1)	5 (5.5)	3 (5.7)
Private (foundation)	193 (12.1)	81 (10.8)	97 (13.8)	8 (8.8)	7 (13.2)
Other	32 (2.0)	13 (1.7)	14 (2.0)	3 (3.3)	2 (3.8)
No funding	248 (15.5)	130 (17.3)	100 (14.2)	11 (12.1)	7 (13.2)
Not reported	543 (34.0)	281 (37.3)	215 (30.7)	29 (31.9)	18 (34.0)
Review is an update, n (%)	32 (2.0)	18 (2.4)	11 (1.6)	2 (2.2)	1 (1.9)
Existing a-priori protocol for the review^d^, n (%)					
Yes (reported)	246 (15.4)	130 (17.3)	90 (12.8)	16 (17.6)	10 (18.9)
No (reported)	16 (1.0)	7 (0.9)	8 (1.1)	1 (1.1)	0 (0.0)
Not mentioned	1336 (83.6)	616 (81.8)	603 (86.0)	74 (81.3)	43 (81.1)
Registration of the review^d^, n (%)					
Yes (reported)	111 (6.9)	59 (7.8)	35 (5.0)	9 (9.9)	8 (15.1)
No (reported)	24 (1.5)	9 (1.2)	12 (1.7)	3 (3.3)	0 (0.0)
Not mentioned	1463 (91.6)	685 (91.0)	654 (93.3)	79 (86.8)	45 (84.9)

^a^Based on the classification system previously suggested by Page et al. [9]

^b^Journals that do not fit in the other categories (e.g., general science journals like PLoS ONE)

^c^Total is greater than 100% because some reviews report multiple sources of funding

^d^As reported by the review authors; we did not search for further evidence of a protocol or registration

**Table 2:** Included study characteristics for non-Cochrane child-relevant systematic reviews published in 2014, overall and by type of review question

Included study characteristics	Overall (*n* = 1598)	Type of review question^a^			
		Therapeutic (*n* = 753)	Epidemiology (*n* = 701)	Diagnostic/ Prognostic (*n* = 91)	Other (*n* = 53)
Study designs: eligible, n (%)					
Only RCTs	203 (12.7)	202 (26.8)	1 (0.1)	0 (0.0)	0 (0.0)
RCTs and other designs	765 (47.9)	476 (63.2)	240 (34.2)	31 (34.1)	18 (34.0)
Only non-RCTs	472 (29.5)	49 (6.5)	346 (49.4)	48 (52.7)	29 (54.7)
Unclear or not reported	158 (9.9)	26 (3.5)	114 (16.3)	12 (13.2)	6 (11.3)
Study designs: included in the review, n (%)					
Only RCTs	231 (14.5)	227 (30.1)	1 (0.1)	0 (0.0)	0 (0.0)
RCTs and other designs	420 (26.3)	334 (44.4)	66 (9.4)	13 (14.3)	7 (13.2)
Only non-RCTs	796 (49.8)	169 (22.4)	523 (74.6)	71 (78.0)	36 (67.9)
Not applicable (empty)	4 (0.3)	4 (0.5)	0 (0.0)	0 (0.0)	0 (0.0)
Unclear or not reported	147 (9.2)	19 (2.5)	111 (15.8)	7 (7.7)	10 (18.9)
Included studies, median (IQR)	19 (11, 33)	15 (9, 26)	23 (13, 40)	18 (12, 28)	20 (12, 41)
Reported on the number of participants, n (%)^b^	692 (43.4)	370 (49.4)	273 (38.9)	41 (45.1)	8 (15.1)
Included participants, median (IQR)	2134 (648, 13,079)	1042 (427, 3324)	11,038 (2098, 63,897)	1086 (515, 2831)	2036 (1284, 5374)
Participant type					
Children only	628 (39.3)	353 (46.9)	212 (30.2)	41 (45.1)	25 (47.2)
Children and adults	787 (49.2)	335 (44.5)	384 (54.8)	46 (50.5)	27 (50.9)
Pregnancy	176 (11.0)	65 (8.6)	99 (14.1)	4 (4.4)	0 (0.0)
Adults only^c^	7 (0.4)	0 (0.0)	6 (0.9)	0 (0.0)	1 (1.9)

*IQR* interquartile range

^a^Based on the classification system previously suggested by Page et al. [9]

^b^The denominator excludes empty reviews which included no participants (*n* = 4, all therapeutic)

^c^ These studies were relevant to child health but included adults only (e.g., outcomes in adults related to an exposure in childhood; intended to include both children and adults but no studies with children were found)

**Table 3:** Methodological characteristics for non-Cochrane child-relevant systematic reviews published in 2014, overall and by type of review question

Methodological characteristics	Overall (*n* = 1598)	Type of review question^a^			
		Therapeutic (*n* = 753)	Epidemiology (*n* = 701)	Diagnostic/ Prognostic (*n* = 91)	Other (*n* = 53)
Objective stated, n (%)	1587 (99.3)	750 (99.6)	694 (99.0)	90 (98.9)	53 (100.0)
Primary outcome(s) specified, n (%)	374 (23.4)	281 (37.3)	74 (10.6)	17 (18.7)	2 (3.8)
When not stated, outcomes of interest can be inferred, n (%)	1099 (89.8)	405 (85.8)	580 (92.5)	65 (87.8)	49 (96.1)
Quality of included studies assessed^b^, n (%)	953 (59.6)	532 (70.7)	325 (46.4)	63 (69.2)	33 (62.3)
Certainty of the body of evidence assessed using GRADE^b^, n (%)					
Yes	72 (4.5)	58 (7.7)	12 (1.7)	2 (2.2)	0 (0.0)
Used another method	30 (1.9)	17 (2.3)	8 (1.1)	1 (1.1)	4 (7.5)
No	1492 (93.4)	674 (89.5)	681 (97.1)	88 (96.7)	49 (92.5)
Evidence synthesis method, n (%)					
Narrative only	741 (46.4)	352 (46.7)	307 (43.8)	36 (39.6)	46 (86.8)
Statistical	857 (53.6)	401 (53.3)	394 (56.2)	55 (60.4)	7 (13.2)
When synthesized statistically, analysis method used, n (%)					
Meta-analysis	827 (96.5)	384 (95.8)	387 (98.2)	50 (90.9)	6 (85.7)
Network meta-analysis	5 (0.6)	5 (1.2)	0 (0.0)	0 (0.0)	0 (0.0)
Individual patient data meta- analysis	12 (1.4)	8 (2.0)	0 (0.0)	4 (7.3)	0 (0.0)
Other^c^	13 (1.5)	4 (1.0)	7 (1.8)	1 (1.8)	1 (14.3)

*GRADE* Grading of Recommendations Assessment, Development and Evaluation [43]

^a^Based on the classification system previously suggested by Page et al. [9]

^b^Denominator excludes empty reviews (*n* = 4, all categorized as therapeutic)

^c^Any other form of analysis (e.g., mathematical modelling, regression)

The SRs spanned 47 of the 53 CRGs and the 8 most populated review groups contained 53.2% (*n* = 849) of the SRs: Developmental, Psychosocial and Learning Problems (*n* = 216, 13.5%); Metabolic and Endocrine Disorders (*n* = 132, 8.3%); Pregnancy and Childbirth (*n* = 124, 7.8%); Common Mental Disorders (*n* = 110, 6.9%); Airways (*n* = 70, 4.4%); Anaesthesia, Critical and Emergency Care (*n* = 69, 4.3%); Public Health (*n* = 67, 4.2%); and Infectious Diseases (*n* = 61, 3.8%). Some notable CRGs containing very few SRs were Skin (*n* = 15, 0.9%), Tobacco Addiction (*n* = 15, 0.9%), Back and Neck (*n* = 14, 0.9%), Neonatal (*n* = 14, 0.9%; neonatal reviews of RCTs only), Eyes and Vision (n = 12, 0.8%), Haematological Malignancies (*n* = 9, 0.6%), Neuromuscular (*n* = 7, 0.4%), and Urology (*n* = 3, 0.2%).

Most often, the SRs synthesized therapeutic (*n* = 753, 47.1%) or epidemiologic (*n* = 701, 43.8%) evidence. Although 39.3% (*n* = 628) of the SRs included evidence specific to children only, fewer were published in pediatric-specific journals (*n* = 283, 17.7%). Approximately half of SRs (*n* = 807, 50.5%) declared at least one external source of support; 15.5% (*n* = 248) had no external funding. The most common sources of support were governments (*n* = 557, 34.9%), academic or research institutes (*n* = 228, 14.3%), and foundations (*n* = 193, 12.1%).

Only three SRs (< 0.01%) included all of the methodological and reporting characteristics that we assessed. Few mentioned a published or registered protocol (*n* = 246, 15.4%) or SR registration (*n* = 111, 6.9%). One-third neglected to mention sources of support (*n* = 543, 34.0%) and more than half did not indicate the total number of included human participants (*n* = 902, 56.6%). Most (*n* = 1224, 76.6%) did not specify one or more primary outcome(s). In 9.9% (*n* = 158) of cases, eligible primary study designs were not mentioned, and in 9.2% (*n* = 147) the type(s) included in the SR was not discernable. Few SRs were identified as updates (*n* = 32, 2.0%). Some were labelled as updates by their authors, but appeared to be new.

Authors rarely searched only for RCTs (*n* = 203, 12.7%). Most commonly, both RCTs and other primary study designs were eligible (*n* = 765, 47.9%). This was reflected in the included primary studies, where most SRs relied solely on evidence from non-RCTs (*n* = 796, 49.8%) or a mix of designs (*n* = 420, 26.3%). Less than two-thirds of SRs (*n* = 953, 59.6%) included an assessment of the quality of the included studies and very few assessed the certainty of the body of evidence (*n* = 102, 6.4%; using Grading of Recommendations Assessment, Development and Evaluation (GRADE) (*n* = 72, 4.5%) or another method (*n* = 30, 1.9%)). The synthesis method used in SRs was roughly equally split between narrative (*n* = 741, 46.4%) and statistical (*n* = 857, 53.6%). There were 5 network meta-analyses and 12 individual patient data meta-analyses.

### Characteristics of the sample by type of review question

Epidemiological SRs included the largest number of primary studies (median (IQR), 23 (13 to 40)) and human participants (11,038 (2098 to 63,897)). Compared to the overall sample, a larger proportion of diagnostic/prognostic SRs were published in Asia (*n* = 25/91, 27.5%), and therapeutic SRs in North America (*n* = 287/753, 38.1%). The Injuries CRG was the most strongly represented group among diagnostic/prognostic SRs, while the Developmental, Psychosocial, and Learning Problems CRG was predominant across all other review types.

Compared to the overall sample, epidemiological SRs more often included mixed samples of children and adults (*n* = 384/701, 54.8%) and less often included only children (*n* = 212/701, 30.2%), but the type of journal in which they were published did not differ. In contrast, the ‘other’ SRs were more often published in pediatric journals (*n* = 16/53, 30.1%), though the sample of these was small. The highest proportion of unfunded SRs was in the therapeutic group (*n* = 130/753, 17.3%). Industry funding was most common among the diagnostic/prognostic (*n* = 5/91, 5.5%) and ‘other’ (*n* = 3/53, 5.7%) SRs.

Reporting of the items of interest that we collected was most complete for the therapeutic SRs and least for the epidemiological and ‘other’ SRs. The therapeutic SRs most often indicated the type of primary studies sought (*n* = 727/753, 96.5%) and included (*n* = 734/753, 97.5%), reported the total number of human participants (*n* = 370/753, 49.4%), and identified a primary outcome (*n* = 281/753, 37.3%). The epidemiological SRs least often identified a published or registered SR protocol (*n* = 90/701, 12.8%) or registration in a SR registry (*n* = 35/701, 5.0%), and most often failed to indicate the type of primary studies sought (*n* = 114/701, 16.3%) and included (*n* = 111/701, 15.8%). The total number of participants (*n* = 273/701, 38.9%) and primary outcome (*n* = 74/701, 10.6%) were relatively infrequently reported among epidemiologic SRs, though the ‘other’ SRs also performed poorly in this respect.

All but one of the SRs seeking to include only RCTs was therapeutic; the epidemiologic, diagnostic/prognostic, and ‘other’ SRs more commonly sought only non-RCTs (49.4%, 52.7%, 54.7%, respectively). Non-RCTs were eligible for inclusion among 69.7% (*n* = 525/753) of the therapeutic SRs. The quality of the included studies was least frequently assessed in the epidemiologic SRs (*n* = 325/701, 46.4%), and most frequently in therapeutic (*n* = 532/753, 70.7%) and diagnostic/prognostic (*n* = 63/91, 69.2%) SRs. Certainty of the body of evidence was most often assessed in therapeutic SRs (*n* = 75/753, 10.0%), and rarely in epidemiologic (*n* = 20/701, 2.8%) or diagnostic/prognostic (*n* = 3/91, 3.3%) SRs.

## Discussion

Though Cochrane SRs are a go-to source for high quality evidence in pediatrics, clinicians must also look elsewhere for evidence on topics that Cochrane reviews do not cover (e.g., epidemiology, and to a lesser extent diagnosis/prognosis). Our findings indicate that outside of the Cochrane Library, an abundance of child-relevant SRs are being produced (i.e., 1598 in 1 year), with a breadth of coverage across almost all CRGs. It appears, however, that publication quantity has outpaced quality. We applied strict inclusion criteria, and sifted through numerous records to identify those that met the minimum standards required to be considered ‘true SRs’ (i.e., the poorest quality reviews would have been excluded). [10, 22] Nevertheless, methodological and reporting deficits among the items that we assessed were prevalent. In the absence of a central repository for high quality child-relevant SR evidence, efforts to ensure that unbiased and accurate child-relevant SRs are readily available and easily identifiable by decision makers are required.

Within Cochrane, the ‘List Project’ is one initiative aiming to align SR priority topics with those that are most likely to impact worldwide health outcomes. [32] To roughly estimate whether the topics covered in non-Cochrane SRs might be aligned with disease burden priorities, we compared the number of publications in various topic areas to the global burden of disease. Similar to child-relevant SRs published in the Cochrane Library, [21] our findings suggest that SRs published outside of the Cochrane Library are diverse but their focus is not necessarily aligned with the global disease burden. Seventy-eight percent of child mortality in 2013 was attributable to lower respiratory infections, other infectious disease (e.g., diarrheal diseases, malaria), conditions related to pregnancy and childbirth, and road injuries. [33] Thus, we would expect the proportion of SRs falling in the Acute Respiratory Infections (*n* = 42, 2.6%), Infectious Diseases (*n* = 61, 3.8%) and Injuries (*n* = 43, 2.7%) CRGs to be higher. Though developing countries face the highest burden of child mortality, [29] few SRs originated from low or lower-middle income countries (*n* = 34, 2.1%). We acknowledge that the burden of disease is only one factor among many (e.g., political or social context, research capacity, resources) that might influence the topics of published systematic reviews. However, a recommended practice to improve the relevance of SRs would be to conduct prioritization exercises involving all key stakeholders (e.g., patients, researchers, decision makers) [34, 35] and to make the results available to those involved in SR conduct.

While the Cochrane Library is known as the go-to source for high quality evidence syntheses, our sample indicates that SRs published elsewhere can be complementary. While Cochrane SRs have focused on healthcare interventions, [10] and more recently diagnostic accuracy, [21] our non-Cochrane sample included a greater focus on epidemiological (43.8%) and diagnostic/prognostic reviews (5.7%). Similarly, while Cochrane SRs rely heavily on evidence from randomised controlled trials (RCTs), [21] even among the therapeutic reviews in our sample, only 30% restricted their synthesis to RCTs. It has been argued that the inclusion of diverse designs in SRs may not only be desirable, but necessary to accommodate the full breadth of clinical research questions and outcomes. [36] One concern though, is that assessing the quality of non-RCTs is a complex task, and only 55.7% (*n* = 759/1363) of the SRs that included non-RCTs in our sample conducted any type of quality assessment. There remains a lack of clarity about the credibility of the results of these studies, and their impact on the quality of the body of evidence.

Poor quality evidence syntheses contribute little to advancing research and practice. Though we did not assess all aspects of reporting endorsed by PRISMA, [11, 22] this descriptive analysis, in which we appraised discrete reporting items, adds to numerous studies across various fields of research showing that adherence to widely endorsed reporting guidance (e.g., PRISMA) [8, 11, 22] is often poor. [37] Contributors to poor reporting quality can include inadequate understanding of the guidance, peer reviewers who are unaware of how to appraise reporting quality, or insufficient scrutiny by editorial boards. Indeed, in characterizing biomedical SRs, Page et al. (2016) [9] found that many authors misinterpreted the PRISMA guidelines. The high prevalence of reporting and methodological deficits, based on the few items that we assessed among our sample, speaks to the difficulty that knowledge users may face when attempting to find high quality evidence syntheses outside of the Cochrane Library. The findings further confirm the need for (a) improved dissemination of these guidelines in ways that are easily understood, and (b) increased oversight by editorial organizations.

Decision-makers rely on unbiased and accurate findings from SRs, and must search beyond the Cochrane library to answer clinical questions for which Cochrane SRs are unavailable. Our analysis indicates that even among those with expertise in evidence synthesis, high quality SRs published outside of the Cochrane Library are challenging and time consuming to find. Despite using a validated search filter, [28] we spent considerable time sifting through records indexed as ‘systematic reviews’ that in reality did not meet the minimum SR criteria, as defined by Cochrane. [10] Only a handful of SRs in the final sample included all of the methodological and reporting features that might be expected (as informed by the aspects of PRISMA reporting standards that we assessed). [11, 22] Available tools to appraise the quality of evidence syntheses [38] require expertise to use and cannot be quickly nor easily applied in practice. With limited guidance available outside of Cochrane, [39, 40] SR updates are difficult for authors to design and publish, and can be challenging for readers to identify. The end result is an increasingly overwhelming, potentially redundant, and confusing body of research that cannot easily be used.

### Strengths and limitations

We have identified key areas in need of improvement in the methodology and reporting of child-relevant SRs. This assessment was based on several reporting standards of interest, but we did not formally appraise the SRs against specific guidance for methods or reporting. In the future, an in-depth characterization of the included SRs against established guidance (e.g., PRISMA, [11, 22] AMSTAR 2 [41]) would add value. One limitation of our sample is that it included only English-language SRs of quantitative data published in 1 year. Other types of reviews fell outside the scope, and SR topics will vary over time. Related to the inconsistent, and overall poor reporting, it was often difficult to extract key information from the SRs. Also, one reviewer extracted data from the SRs, which increased the risk of error compared to duplicate extraction. [42] We mitigated these risks by pilot testing the data extraction form, meeting regularly to address concerns, and re-evaluating data items with poor agreement between reviewers. As we did not seek additional information about the SRs, it was not possible to separate methodological from reporting deficits.

## Conclusion

Knowledge users require high quality pediatric-specific evidence to guide decision-making in child health. Though Cochrane SRs remain the go-to source for high quality SRs, knowledge users may search elsewhere for evidence syntheses that suit their needs. We have shown that non-Cochrane evidence syntheses may complement those within the Cochrane Library (e.g., greater focus on epidemiological reviews). However, a high prevalence of methodological and reporting shortfalls means that finding high quality evidence syntheses outside of the Cochrane Library may not be easy nor practical. Improvement is needed to ensure easy access to high quality evidence syntheses outside of the Cochrane Library.

## Additional files

Additional file 1: Search strategy. This file documents the search strategy used to locate included studies (PDF 193 kb)

Additional file 2: Data extraction guide for a descriptive analysis of non-Cochrane child-relevant systematic reviews. This file provides a detailed description of the data extracted for analysis. (PDF 423 kb)

## Abbreviations

GRADE: Grading of Recommendations Assessment Development and Evaluation
IQR: inter-quartile range
RCT: randomised controlled trial
SR: systematic review
