# Supplementary material for: A descriptive analysis of non-Cochrane child-relevant systematic reviews published in 2014
Source: BMC Med Res Methodol. 2018 Oct 1;18:99. doi: 10.1186/s12874-018-0562-2 (PMC6167827; doi:10.1186/s12874-018-0562-2)
Supplement: Supplementary file 1 — Search strategy. This file documents the search strategy used to locate included studies (PDF 193 kb) [file 12874_2018_562_MOESM1_ESM.pdf]

Supplementary file 1. literature search strategy

Database: Ovid MEDLINE(R) In-Process & Other Non-Indexed Citations and Ovid MEDLINE(R) 1946 to Present

1. exp Adolescent/
2. exp Child/
3. exp Infant/
4. exp Minors/
5. exp Pediatrics/
6. exp Puberty/
7. exp Schools/
8. (baby* or babies or infant* or infancy or neonat* or newborn* or postmatur* or prematur* or preterm*).mp.
9. (boy* or girl* or teen*).mp.
10. (child* or kid or kids or preschool* or school age* or schoolchild* or toddler*).mp.
11. (elementary school* or high school* or highschool* or kindergar* or nursery school* or primary school* or secondary school*).mp.
12. minors*.mp.
13. (paediatric* or peadiatric* or pediatric*).mp.
14. (prepubescen* or pubescen* or pubert*).mp.
15. or/1-14 [Combined MeSH & Keywords for children] (3712156)
16. medline.tw
17. systematic review.tw
18. meta analysis.pt
19. or/16-18 [HIRU SR filter for max specificity] (141272)
20. and/15,19 [Combined child terms and SR filter] (25710)
21. Cochrane database of systematic reviews.jn [Journal name] (11759)
22. 20 not 21 [removing Cochrane SRs from the search results] (22577)
23. exp animals/ not humans.sh.
24. 22 not 23 [removing animal studies from the search results] (22526)
25. limit 24 to yr="2014" [publication date limit] (2901)
26. limit 25 to english (2824)
27. remove duplicates from 26 (2546)

Database: CINAHL via EBSCOhost

S1 (MH "Adolescence+")
S2 (MH "Child+")
S3 (MH "Infant+")
S4 (MH "Minors (Legal)")
S5 (MH "Pediatrics+")
S6 (MH "Puberty+")
S7 adoles*

S8 baby* or babies or infant* or infancy or neonat* or newborn* or postmatur* or prematur* or preterm*
S9 boy* or girl* or teen*
S10 child* or kid or kids or preschool* or “school age*” or schoolchild* or toddler*
S11 “elementary school*” or “high school*” or highschool* or kindergar* or “nursery school*” or “primary school*” or “secondary school*”
S12 minors*
S13 paediatric* or peadiatric* or pediatric*
S14 prepubescen* or pubescen* or pubert*
S15 S1 OR S2 OR S3 OR S4 OR S5 OR S6 OR S7 OR S8 OR S9 OR S10 OR S11 OR S12 OR S13 OR S14 [Combined CINAHL Headings and keywords for children] (796,970)
S16 TX meta analysis
S17 PT systematic review
S18 S16 OR S17 [SR filter] (112,570)
S19 S15 AND S18 [Child results limited to SRs] (23,259)
S20 SO cochrane database of systematic reviews
S21 S19 NOT S20 [Removal of Cochrane SRs] (21,334)
S22 (MH "Animals+") not (MH "Humans")
S23 S21 NOT S22 [removal of animal studies] (21,256)
S24 S21 NOT S22 Published Date: 20140101-20141231 [publication date limit] (2431)
S25 S21 NOT S22 English Language ; Published Date: 20140101-20141231 [language limit] (2378)

Database 3: Web of Science Core Collection

NOTE: All searches limited to 2014
1. TS=(baby or babies or infant* or neonat* or toddler* or child* or adolescen* or teen* or youth or p?ediatric*) [topic search for children] (83114)
2. TS=((meta analys*) OR meta-analys* OR metaanalys* OR (systematic review*)) (29629)
3. #2 AND #1 [Child SRs] (3605)
4. SO=(Cochrane Database of Systematic Reviews) (826)
5. #3 NOT #4 [remove Cochrane SRs] (1499)
6. TS=(animal* or bovine or cat* or dog* or mice or mouse or pig* or porcine or rat or zebrafish) NOT TS=(human*)
7. #5 NOT #6 [animal filter] (3209)
8. #5 NOT #6 Refined by: DOCUMENT TYPES: (REVIEW) (1135)
9. #5 NOT #6 Refined by: DOCUMENT TYPES: (REVIEW) AND LANGUAGES: (ENGLISH) (1116)

Database 4: PubMed

((((Infant[MeSH] OR Infant* OR infancy OR Newborn* OR Baby* OR Babies OR Neonat* OR Preterm* OR Prematur* OR Postmatur* OR Child[MeSH] OR Child* OR Schoolchild* OR School age* OR Preschool* OR Kid OR kids OR Toddler* OR Adolescent[MeSH] OR Adoles* OR Teen* OR Boy OR Boys OR Girl* OR Minors[MeSH] OR Minors* OR Puberty[MeSH] OR

Pubert* OR Pubescen* OR Prepubescen* OR Pediatrics[MeSH] OR Pediatric* OR Paediatric* OR Peadiatric* OR Schools[MeSH] OR Nursery school* OR Kindergar* OR Primary school* OR Secondary school* OR Elementary school* OR High school* OR Highschool*) AND (MEDLINE[Title/Abstract] OR (systematic[Title/Abstract] AND review[Title/Abstract]) OR meta analysis[Publication Type])) NOT (((Animals[MESH] OR Animal Experimentation[MESH] OR "Models, Animal"[MESH] OR Vertebrates[MESH]) NOT (Humans[MESH] OR Human experimentation[MESH])) OR (((animals[tiab] OR animal model[tiab] OR rat[tiab] OR rats[tiab] OR mouse[tiab] OR mice[tiab] OR rabbit[tiab] OR rabbits[tiab] OR pig[tiab] OR pigs[tiab] OR porcine[tiab] OR swine[tiab] OR dog[tiab] OR dogs[tiab] OR hamster[tiab] OR hamsters[tiab] OR chicken[tiab] OR chickens[tiab] OR sheep[tiab]) AND (publisher[sb] OR inprocess[sb] OR pubmednotmedline[sb])) NOT (human[ti] OR humans[ti] OR people[ti] OR children[ti] OR adults[ti] OR seniors[ti] OR patient[ti] OR patients[ti])))) NOT ("the cochrane database of systematic reviews"[Journal])) AND ("2014/01/01"[PDat] : "2014/12/31"[PDat])
Filters: English
