# Supplementary material for: A descriptive analysis of non-Cochrane child-relevant systematic reviews published in 2014
Source: BMC Med Res Methodol. 2018 Oct 1;18:99. doi: 10.1186/s12874-018-0562-2 (PMC6167827; doi:10.1186/s12874-018-0562-2)
Supplement: Supplementary file 2 — Data extraction guide for a descriptive analysis of non-Cochrane child-relevant systematic reviews. This file provides a detailed description of the data extracted for analysis. (PDF 423 kb) [file 12874_2018_562_MOESM2_ESM.pdf]

Supplemental file 2. Data extraction guide for a descriptive analysis of non-Cochrane child-relevant systematic reviews

Field	Response Options	Instructions
**REVIEW CHARACTERISTICS**		
**Country (corresponding author)**	Open text	Abbreviate United States as USA; United Kingdom as UK. Omit 'the' (e.g., just enter in 'Netherlands').
**Journal type**	□General medical journal<br>□Specialty medical journal<br>□General pediatric journal<br>□Specialty pediatric journal<br>□Other	**General medical:** e.g., Lancet, BMJ, JAMA, CMAJ, NEJM<br>**Specialty medical:** e.g., Journal of Clinical Oncology, Circulation<br>**General pediatric:** e.g., Pediatrics, Archives of Pediatrics & Adolescent Medicine<br>**Specialty pediatric:** e.g., Pediatric Emergency Care, Journal of Pediatric Orthopedics<br>**Other:** the journal does not fit in one of the above categories, e.g., general science journals such as PLOS ONE
**Clinical area: based on Cochrane Review Groups**	1. Acute Respiratory Infections Group<br>2. Airways Group<br>3. Anaesthesia, Critical and Emergency Care Group<br>4. Back and Neck Group<br>5. Bone, Joint and Muscle Trauma Group<br>6. Breast Cancer Group<br>7. Childhood Cancer Group<br>8. Colorectal Cancer Group<br>9. Common Mental Disorders Group<br>10. Consumers and Communication Group<br>11. Cystic Fibrosis and Genetic Disorders Group<br>12. Dementia and Cognitive Improvement Group<br>13. Developmental, Psychosocial and Learning Problems Group<br>14. Drugs and Alcohol Group<br>15. Effective Practice and Organisation of Care Group	What Cochrane Review Group (CRG) would this review likely fall under? Review the topics of the 53 CRGs (hyperlinked in the Response column), and make your choice.<br><br>Highlight for discussion if it is too difficult or if it seems like the review could fit in more than one group, **write key words in the next column**.<br><br>**Note:** consult the help document for examples of topics in each group.

Field	Response Options	Instructions
	16. ENT Group<br>17. Epilepsy Group<br>18. Eyes and Vision Group<br>19. Fertility Regulation Group<br>20. Gynaecological, Neuro-oncology and Orphan Cancer Group<br>21. Gynaecology and Fertility Group<br>22. Haematological Malignancies Group<br>23. Heart Group<br>24. Hepato-Biliary Group<br>25. HIV/AIDS Group<br>26. Hypertension Group<br>27. IBD Group<br>28. Incontinence Group<br>29. Infectious Diseases Group<br>30. Injuries Group<br>31. Kidney and Transplant Group<br>32. Lung Cancer Group<br>33. Metabolic and Endocrine Disorders Group<br>34. Methodology Review Group<br>35. Movement Disorders Group<br>36. Multiple Sclerosis and Rare Diseases of the CNS Group<br>37. Musculoskeletal Group<br>38. Neonatal Group<br>39. Neuromuscular Group<br>40. Oral Health Group<br>41. Pain, Palliative and Supportive Care Group<br>42. Pregnancy and Childbirth Group<br>43. Public Health Group<br>44. Schizophrenia Group	

Field	Response Options	Instructions
	45. Skin Group<br>46. STI Group<br>47. Stroke Group<br>48. Tobacco Addiction Group<br>49. Upper GI and Pancreatic Diseases Group<br>50. Urology Group<br>51. Vascular Group<br>52. Work Group<br>53. Wounds Group	
**Key words (if clinical area unclear)**	Open text	**This column only needs to be used if you cannot decide on a CRG.** Extract key words relating to the clinical area.<br>**Example:** “Disclosure and non-disclosure of concussion and concussion symptoms in athletes: Review and application of the socio-ecological framework”. Key words provided by the authors included: Policy, reporting, sports, traumatic brain injury.<br><br>Since the purpose of extracting key words is to easier identify a CRG the review would likely fall under, here we would most likely want to extract “traumatic brain injury”.
**Type of a review question**	□Therapeutic<br>□Epidemiology<br>□Diagnosis/Prognosis<br>□Other<br><br>(Based on the work of Page et al., 2016) [1]	**Therapeutic:** Includes treatment and prevention (interventions would typically fall in this group)<br>**Epidemiology:** Includes prevalence reviews and those looking at the association between an exposure and an outcome (e.g., studies of etiology)<br>**Diagnosis/prognosis:** reviews of diagnostic test accuracy (e.g., sensitivity, specificity, false +/-), clinical prediction rules<br>**Other:** psychometric properties (e.g., reliability and validity) of tools, cost of illness, and other topics that would not fit into the other categories
**Was the review an update?**	□ Yes<br>□ No	**Yes:** the authors identify the review as an update of an existing review. Authors must have incorporated the data from the previous

Field	Response Options	Instructions
		review, not conducted a whole new review including only new evidence.<br>**No:** the authors identify the review as a new review/do not mention that it is an update.
**Was the funding source specified?**[b]	□Yes<br>□No	**Yes:** the authors specified the funding source, or that the review was unfunded (could include personal funding).<br>**No:** the authors make no mention of funding source.<br>**Note:** A funding-specific statement is required; stating no conflicts of interest not adequate to say the review was unfunded.
**Who funded the review?**[b]	□Government<br>□Academic or research institute<br>□Private<br>□Industry<br>□No external funding<br>□Other (specify in Comments)<br><br>(Informed by Klassen et al. 2002)[2]	**Government:** Includes Canadian Institutes of Health Research, National Institutes of Health, and other similar funding sources<br>**Academic or Research Institute:** e.g., hospital research institutes, university funding<br>**Private:** when a foundation (or trust) is listed as the source of funding<br>**Industry:** the review received industry funding (e.g., a pharmaceutical company)<br>**No external funding:** the authors specify that the review was not funded<br>**Other:** all UN agencies, WHO, or other funding sources that do not fit in other categories<br>**Note:** Each funding type only needs to be listed once, even if the review was funded by multiple of the same type of funding source. Consult the help document for examples of which category various funding sources would fit in.
**Who funded the review? (2,3,4)**[b]	□Government<br>□Academic or research institute<br>□Private<br>□Industry<br>□No external funding<br>□Other (specify in Comments)<br>□NA	Indicate other sources of funding if the review was funded by more than one source. Please choose the highest menu item first, then go through the menu until all funding sources are listed.<br>If there was only one source of funding, **choose NA** for this column.
**Existence of an a-priori protocol**[a,b]	□Yes<br>□No	**Yes:** the authors indicated that a protocol was developed a priori.<br>**No:** the authors indicated that protocol was not developed a priori.

Field	Response Options	Instructions
	□Not mentioned	**Not mentioned**: the authors do not mention whether a protocol was developed.<br>**Note:** If mentioned that the review was registered in PROSPERO, this means there is a protocol
**Registration of the review**[a,b]	□Yes<br>□No<br>□Not mentioned	**Yes**: the authors indicated that a review was registered, or indicate that the protocol was registered (which implies registration of the review)<br>**No:** the authors indicated that a review was not registered.<br>**Not mentioned**: the authors did not mention whether a review was registered.
**CHARACTERISTICS OF INCLUDED STUDIES**		
**Study designs sought** (part of eligibility criteria[a,b])	□Only RCTs<br>□Only non-RCTs<br>□RCTS and other designs<br>□Unclear/unreported	**Only RCTs:** randomized controlled trials (parallel group or crossover designs)<br>**Only non-RCTs (other designs):** e.g., observational or epidemiological studies – cohort, cross sectional, before-after, case-control, time series<br>**RCTs and other designs:** Searched for any study design (this should be stated – e.g., searched for 'all studies' on a particular topic). Also, e.g., if they only excluded case control studies or reviews, you can choose this as they can be assumed to have included all other designs<br>**Unclear/unreported:** there is no mention of what type of design was sought – e.g., searched for 'studies' with no mention of design<br>**Note:** Do not guess or attempt to infer, choose unclear whenever the type of design sought (or excluded) is not clearly stated
**Study designs included** (part of adequate description of included studies[a,b])	□Only RCTs<br>□Other designs<br>□RCTS and other designs<br>□Unclear/unreported	**As above**<br>**Note:** Do not guess or attempt to infer, choose unclear whenever the type of designs obtained are not clearly stated (e.g., in results text or tables). Do not search reference lists.
**Intended type of participants included in the review?** (part of eligibility criteria[a,b])	□Children only<br>□Children and adults<br>□Adults<br>□Pregnancy	**Children only**: Children are defined as individuals aged 0 to 18. Use this definition, rather than what might be used in the SR.

Field	Response Options	Instructions
		**Children and adults:** If population in the review included children and adults. If any participants are >18 years (e.g., 0-20 years), choose this category.<br>**Adults:** If population included only adults, but the review question is relevant to children (e.g., family interventions, nurse involvement in pediatric care if an outcome of interest is relevant to children, etc.)<br>**Pregnancy:** e.g. include (but are not limited to): a SR assessed outcomes on newborns as a consequence of exposure or intervention for the mothers, breastfeeding.
**Number of reports of studies included in the review**[a] (flow of records[a])	Number	Enter the number of citations included in the review (e.g., if there were 9 reports on 7 studies, enter '9'). This can typically be found in the text of the methods or results, or in a PRISMA flow chart. Enter whole numbers ≥0.<br>**Note:** if total number of studies is not reported, the review should have been excluded for not meeting the criteria for an SR (do not extract data, flag for exclusion)
**Was the number of participants explicitly reported?** (part of adequate description of included studies[a,b])	□Yes<br>□No	**Yes**: the authors explicitly report on the total number of the participants included (or a summary of groups, if there was a comparison of 2+ groups such as intervention and control). This can typically be found in the results, and sometimes a PRISMA chart. Please calculate ONLY IF a summary value is given (e.g., for two groups such as control vs. treatment).<br>**No**: the number of the participants is not obviously reported. DO NOT calculate across included studies.
**Total number of participants in the review**	Number	If a review's population members of naturally occurring groups, such as families or classes, enter the number of individuals not the groups<br>If a number was given as an estimate (e.g. >2 million, or approximately 1 million, etc.), enter the estimated lower number in digits using no decimals, spaces or commas (e.g. 2000000 or 1000000)<br>**NA:** If there are no studies in the review, the authors cannot report on the number of participants.

Field	Response Options	Instructions
**METHODOLOGICAL APPROACHES**		
**Objective stated**[a,b]	□Yes<br>□No	**Yes:** the authors provide one or more objectives/purpose for the review, even if this is only mentioned in the abstract. Ideally would be based on PICO but this is not a requirement for this project.<br>**No:** no objectives specified anywhere in the paper (before results)
**Primary outcome specified**	□Yes<br>□No	**Yes:** There is a specific statement e.g., "the primary outcome is/was…" – must explicitly state what the primary outcome is (could also call this a dependent variable or something similar) (note: for SRs there can be several primary outcomes, so long as they are identified); just mentioning 'outcomes' is not enough.<br>**No:** There is no explicit statement about the primary outcome (even if there is only 1)
**Outcomes of interest listed or can be inferred**[a,b]	□Yes<br>□No<br>□NA	**Yes:** a-priori outcomes (at least one) of interest are listed or can be inferred from statements in the introduction, objectives, hypotheses or methods (e.g., inclusion/exclusion, data extraction)<br>**No:** Cannot tell the intended outcomes from the introduction, objectives, hypotheses or methods (or intended outcomes are not discrete and instead are only vague categories e.g., 'behavioural outcomes', so you cannot tell what exactly they are looking for)<br>**NA:** If a primary outcome is stated, then this item is not applicable
**Was the quality of included studies formally assessed?**[a,b]	□Yes<br>□No<br>□NA	**Yes**: a tool was used to appraise quality of included studies (e.g., Cochrane risk of bias tool, Newcastle-Ottawa scale, or one developed by the authors for use in the SR)<br>**No**: there was no formal quality appraisal<br>**NA**: no studies were found, thus the quality could not be assessed
**Was the quality of evidence assessed using GRADE?**[a]	□Yes<br>□No<br>□Other method<br>□NA	This question pertains to the ***quality of evidence*** usually assessed by GRADE, and not ***quality of the included studies*** (above).<br>**Yes**: used the Grades of Recommendation, Assessment, Development and Evaluation (GRADE) assessment to grade the quality of the evidence.<br>**No**: the GRADE tool was not used<br>**Other tool:** some tool/method other than GRADE was used to assess the quality of the evidence (specify in comments)

Field	Response Options	Instructions
		**NA**: no studies were found, thus the quality could not be assessed
**How was the evidence synthesized?**	□Narratively only<br>□Statistically	**Narratively only:** results were narratively summarized the findings of multiple studies are explained. Some authors call this a 'qualitative synthesis'. There is no statistical analysis.<br>**Statistically:** results were analyzed using a statistical approach (e.g. meta-analysis or network meta-analysis).
**If the evidence was synthesized statistically, what method was used?**	□Meta-analysis<br>□Network meta-analysis<br>□Individual patient data meta-analysis<br>□NA	Indicate method used from the 3 choices.<br>**Mixed:** multiple methods were used in the review (list in comments)<br>**NA**: the results were not synthesized statistically

[a] The item is a PRISMA reporting item [3, 4]

[b] The item may be used to appraise systematic review quality using AMSTAR 2 [5]

References

[1] Page MJ, Shamseer L, Altman DG, et al. Epidemiology and reporting characteristics of systematic reviews of biomedical research: a cross-sectional study. PLoS Med. 2016;13(5):e1002028. doi: 10.1371/journal.pmed.1002028.

[2] Klassen TP, Wiebe N, Russell K, et al. Abstracts of randomized controlled trials presented at the society for pediatric research meeting: An example of publication bias. Arch Pediatr Adolesc Med. 2002;156(5):474-9.

[3] Liberati A, Altman DG, Tetzlaff J, et al. The PRISMA statement for reporting systematic reviews and meta-analyses of studies that evaluate health care interventions: explanation and elaboration. PLoS Med. 2009;6(7):e1000100. https://doi.org/10.1371/journal.pmed.1000100

[4] Moher D, Tetzlaff J, Tricco AC, Sampson M, Altman DG. Epidemiology and reporting characteristics of systematic reviews. PLoS Med. 2007;4(3):e78.13. https://doi.org/10.1371/journal.pmed.0040078

[5] Shea BJ, Reeves BC, Wells G, Thuku M, Hamel C, Moran J, et al. AMSTAR 2: a critical appraisal tool for systematic reviews that include randomised or non-randomised studies of healthcare interventions, or both. BMJ. 2017;358:j4008.
